# A precise model for skin cancer diagnosis using hybrid U-Net and improved MobileNet-V3 with hyperparameters optimization

**DOI:** 10.1038/s41598-024-54212-8

**Published:** 2024-02-21

**Authors:** Umesh Kumar Lilhore, Sarita Simaiya, Yogesh Kumar Sharma, Kuldeep Singh Kaswan, K. B. V. Brahma Rao, V. V. R. Maheswara Rao, Anupam Baliyan, Anchit Bijalwan, Roobaea Alroobaea

**Affiliations:** 1 Department of Computer Science and Engineering, Chandigarh University, Mohali, Punjab 140413 India; 2 Department of Computer Science and Engineering, Koneru Lakshmaiah Education Foundation, Greenfield, Vaddeswaram, Guntur, AP India; 3 School of Computing Science and Engineering, Galgotias University, Greater Noida, Uttar Pradesh India; 4 Departmentt of Computer Science and Engineering, Shri Vishnu Engineering College for Women (A), Bhimavaram, India; 5 Arba Minch University, Arba Minch, Ethiopia; 6 Department of Computer Science, College of Computers and Information Technology, Taif University, P. O. Box 11099, 21944 Taif, Saudi Arabia

**Keywords:** Health care, Convolution Neural Network, Skin cancer, Transfer learning, U-Net, Deep learning, Cancer imaging, Skin cancer

## Abstract

Skin cancer is a frequently occurring and possibly deadly disease that necessitates prompt and precise diagnosis in order to ensure efficacious treatment. This paper introduces an innovative approach for accurately identifying skin cancer by utilizing Convolution Neural Network architecture and optimizing hyperparameters. The proposed approach aims to increase the precision and efficacy of skin cancer recognition and consequently enhance patients' experiences. This investigation aims to tackle various significant challenges in skin cancer recognition, encompassing feature extraction, model architecture design, and optimizing hyperparameters. The proposed model utilizes advanced deep-learning methodologies to extract complex features and patterns from skin cancer images. We enhance the learning procedure of deep learning by integrating Standard U-Net and Improved MobileNet-V3 with optimization techniques, allowing the model to differentiate malignant and benign skin cancers. Also substituted the crossed-entropy loss function of the Mobilenet-v3 mathematical framework with a bias loss function to enhance the accuracy. The model's squeeze and excitation component was replaced with the practical channel attention component to achieve parameter reduction. Integrating cross-layer connections among Mobile modules has been proposed to leverage synthetic features effectively. The dilated convolutions were incorporated into the model to enhance the receptive field. The optimization of hyperparameters is of utmost importance in improving the efficiency of deep learning models. To fine-tune the model's hyperparameter, we employ sophisticated optimization methods such as the Bayesian optimization method using pre-trained CNN architecture MobileNet-V3. The proposed model is compared with existing models, i.e., MobileNet, VGG-16, MobileNet-V2, Resnet-152v2 and VGG-19 on the “HAM-10000 Melanoma Skin Cancer dataset". The empirical findings illustrate that the proposed optimized hybrid MobileNet-V3 model outperforms existing skin cancer detection and segmentation techniques based on high precision of 97.84%, sensitivity of 96.35%, accuracy of 98.86% and specificity of 97.32%. The enhanced performance of this research resulted in timelier and more precise diagnoses, potentially contributing to life-saving outcomes and mitigating healthcare expenditures.

## Introduction

**Figure 1:**
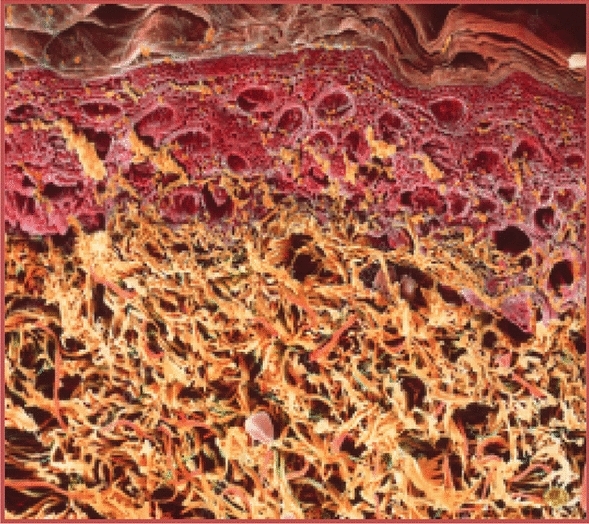
Skin cancer sample^23^.

Skin cancer is considered one of the most widespread and significant forms of cancer globally, with its occurrence consistently rising in recent years. The prompt and precise identification of a medical condition is crucial for successfully implementing treatment strategies and enhancing patient results. Historically, dermatologists have traditionally depended on their professional knowledge and visual examination of skin lesions in order to detect possible malignancies. Nevertheless, this procedure is inherently subjective and prone to diagnostic inaccuracies^1^. In response to all these barriers, Computer-Aided Diagnosis (CAD) frameworks significantly improved medical dermatology. Deep learning models that fall into the category of "convolutional neural networks," also known as "CNNs," have shown remarkable promise for improving the accuracy of skin cancer recognition^2^. Figure 1 presents an image of a skin cancer sample.

Melanoma and non-melanoma are the two primary subtypes of skin cancer, distinguished based on the cell that is transformed into a cancerous form. Various machine learning and deep learning frameworks and methodologies have been suggested to detect, classify, and segment skin cancer. Examples of these include Support Vector Machines (SVM), Decision Trees (DT), Fuzzy C-means, Recurrent Neural Networks (RNN), Convolutional Neural Networks (CNN) and Deep Neural Networks (DNN)^3^. CNN is a widely employed algorithm for automated feature learning and extraction, known for its exceptional performance in detection tasks^4^. Moreover, the training stage for deep learning networks often necessitates a substantial volume of data. Therefore, using transfer learning to refine a pre-existing network on comparable tasks can reduce training complexity, expedite convergence, and decrease training duration^5^.

Additionally, it is possible to utilize pre-trained deep learning models as feature extractors without additional training, provided they have already been trained on tasks or domains that are similar or related^6^. The deep learning model may acquire features that contain noise, thereby impacting the accuracy of the final classification. This can be attributed to the inclusion of non-relevant features and the naturally high dimensionality of the characteristics learned. Therefore, utilizing optimization techniques, specifically metaheuristic methods, can provide a viable approach for selecting elements. This approach involves identifying and choosing the most pertinent features, thereby enhancing the precision of recognition^7^.

This research paper introduces a comprehensive methodology to handle the complex issues related to skin cancer detection. This research presents a novel skin cancer recognition model based on optimized CNN architecture. This research utilizes deep learning and advanced optimization techniques to improve skin cancer detection's precision and effectiveness. The motivation behind this research originates from the critical requirement to deal with the primary healthcare problem related to dermatological cancer. Millions of people worldwide have skin cancer, and the disease's incidence is still rising. The prompt and precise identification of a condition or disease is crucial to administer the correct therapy and, finally, to preserve human life. Conventional diagnostic techniques, dependent on individual observation, are subjective and may change in precision based on the training and experience of the medical professional^8^.

The presence of subjectivity and the possibility of error underscore the utmost significance of advancing the development of more accurate and automated mechanisms for identifying skin cancer. The primary motivation behind our research stems from the pressing demand for enhanced techniques in detecting skin cancer. We harness their capabilities by harnessing deep learning and optimization methodologies to address this objective. Furthermore, the emergence of deep learning methods, i.e., CNNs, has shown the capacity for transformation in analyzing healthcare images. The capability of CNNs to identify complicated characteristics from visuals and to make decisions based on information presents an exciting chance for improvement in the discipline of skin diseases^9^. The convergence of urgent medical necessity and state-of-the-art technology constitutes the primary impetus for our investigation, compelling us to construct a sophisticated model for identifying skin cancer that harnesses the capabilities of deep learning and optimization methodologies.

The primary contributions of our research encompass innovative model architecture, meticulous hyperparameter optimization, persuasive experimental findings, and significant clinical implications. These collective achievements propel the current state of advancement in dermatological examinations and contribute to the enhancement of the treatment of patients. The proposed research presents several significant contributions to the domain of skin cancer detection and healthcare:

- We enhance the learning procedure of deep learning by integrating Standard U-Net and Improved MobileNet-V3 with optimization techniques, which allows the model to accomplish better differentiation among malignant and benign skin cancers.
- Our research focuses on the meticulous examination of hyperparameter optimization, a crucial factor that substantially influences the efficacy of deep learning models. Advanced optimization techniques, i.e., Bayesian optimization and grid search, are utilized to optimize the hyperparameter of the model effectively.
- The optimization of hyperparameters is of utmost importance in enhancing the efficiency of deep learning models. To fine-tune the model's hyperparameter, we employ sophisticated optimization methods such as the Bayesian optimization method using pre-trained CNN architectures, i.e., MobileNet-V3, on the “HAM-10000 Melanoma Skin Cancer” dataset.
- The proposed hybrid MobileNet-V3 model outperforms existing techniques based on high precision of 97.84%, sensitivity of 96.35%, accuracy of 98.86%, and specificity of 97.32%.

The article is organized in the following way: after an introductory part that provides an overview of the motivation and aims, a comprehensive examination of existing literature on skin cancer detection is presented in section two. The methodology section three offers a thorough account of the procedures employed for data collection, preliminary processing, and the development of a hybrid architecture specifically designed to detect skin cancer. Additionally, it includes a detailed explanation of the process of hyperparameter optimization. Section four provides a detailed description of the experimental setup, presents the results and performance indicators obtained, and compares them to existing methods. An extensive examination of the consequences, advantages, and drawbacks of the proposed method follows. The conclusion section five serves the purpose of combining the main findings of the research and highlighting their potential clinical significance.

## Literature review

Previous research was investigated concerning the categorization of Skin Cancer using deep learning techniques. In the context of the categorization of Skin Cancer utilizing deep learning techniques, scholars generated skin lesion images sourced from publicly available websites.

Data augmentation methods were employed alongside five-fold cross-validation methods to enhance the dataset. The investigators conducted experiments to evaluate the performance of pre-trained VGG-16, ResNet50, and InceptionNet-V3 models in classifying Skin Cancer diseases. The ResNet50 model was selected due to its superior accuracy rate compared to the other models. The ResNet50 model achieves an F1 Score of 0.82, as reported in reference^10^. The researchers used the Xception training model with the Grad-Cam and the LIME algorithm techniques to predict skin cancer^11^. The Xception and the DenseNet models have implemented an integrated strategy utilizing unity. The research involved the analysis of the efficiency scores retrieved from experiments conducted on a publicly accessible dataset. These experiments were carried out using this suggested ensemble strategy. The results indicated an average recall, precision, F1 score, and accuracy rate of 86.74%, 85.8%, 86.24%, and 88.93%, respectively.

The article^12^ presents research on the utilization of YOLOv4-DarkNet along with active contour techniques to localize and segment melanoma. The method was evaluated on the International Skin Imaging Collaboration (ISIC) datasets for 2016 and 2018. The values of one and the reported Jaccard coefficient were 1 and 0.989, respectively. The paper^13^ discusses the topology of FC-DPN segmentation. The construction of the network involved the implementation of a dual-path and fully convolutional architecture. In the revised ISIC 2017 test dataset, the suggested approach achieved a Jaccard index of 81.32% and an average dice coefficient of 87.2%.

Similarly, for the PH2 dataset, a Jaccard index of 84.3% and an average dice coefficient of 91.76% were obtained. The paper discusses a CNN model for the classification of skin cancer^14^. The dataset was initially gathered and subsequently categorized into four distinct groups of skin cancer images. Later, augmentation techniques were employed to expand the dataset's size. During the testing phase, the algorithm developed by the researchers achieved an accuracy of 96.28%, surpassing the accuracy of the GoogleNet and MobileNet models by 1.76% and 1.12%, respectively.

The application of deep learning techniques for skin cancer detection has been the subject of considerable investigation in recent academic literature. Several deep learning techniques, including CNNs, have been utilized to categorize skin lesions as either cancerous or non-cancerous precisely. The authors of^15^ present a unique deep learning network called the Dual Optimisation Utilising Deep Learning Network. This network was designed for skin cancer identification using dermoscopic images and achieved an impressive precision of 98.76%. This paper presents a thorough examination of conventional and deep-learning methodologies employed in the prompt identification of skin cancer. It critically evaluates the performance of these techniques and examines the datasets utilized for training and testing purposes^16^. The authors have developed a deep learning system using a CNN to detect melanoma lesions. This system has demonstrated superior performance regarding diagnostic accuracy compared to existing methods, as reported in reference^17^. The research proposes using convolutional-based neural networks with deep reinforcement learning to identify cancer-affected skin. It aims to overcome the challenges related to generalizability by implying the adoption of ensemble models to achieve optimal output, as indicated by reference^18^.

Recent studies have utilized various types of classification and preliminary processing methods to conduct morphological change examines on grey-level skin cancer images. These images were obtained from the PH2 repository and were subjected to classification and clustering procedures using a pre-trained Levenberg-Mean neural network^6^. Transfer learning has been utilized to forecast skin cancer visuals from the HAM10000 database precisely using the MobileNet CNN^19^. The recent research on the application of CNN in predicting skin cancer has identified significant challenges in incorporating the entire spectrum of the patient population and various melanoma subtypes^20^. In addition, ongoing efforts are being made to develop optimization algorithms that enhance accuracy by adjusting hyperparameters^21^. Moreover, recent studies have put forth methods to identify instances of shortcut development during the training of models based on convolutional neural networks on both the ISIC Storage collection. Recent research has also emphasized using comprehensible deep-learning techniques for the multi-class separation and categorization of skin lesion images^22^.

Predicting skin diseases using AI holds enormous promise for better early detection and treatment results^23^. This study offered a new way to improve skin disease prediction models by addressing class imbalance utilizing data balancing using class weighting and transfer learning approaches. They have also investigated the usefulness of data balancing using class weighting to boost TL performance for skin disease prediction. Experiments and performance evaluations have yielded important insights and outcomes, proving that the suggested method works. Optimized kernel-enhanced Resnet-dropped extended short-term memory technique for varicose vein disease detection was addressed in^24^. After processing 11,350 photos of leg veins, the experiment was evaluated using metrics such as accuracy, precision, kappa, mean square error (MSE), and time complexity. With a time complexity of 25.36 ms, a kappa of 95.77%, a precision of 98.69%, and an MSE of 1.73, the suggested model attained an accuracy of 98.5%.

Although skin cancer is dangerous, it is successfully treatable if caught early. A procedure is involved in the suggested approach^25^. Improving the quality and extracting useful information from the raw photos is the first step in the pre-processing phase. The next step is to feed the pre-processed pictures into a GRU Network, a deep-learning model that excels at capturing sequential information. The GRU Network was fine-tuned by the author using an improved version of the Orca Predation Algorithm (OPA). In comparison to previous approaches, the GRU/IOPA system achieved better results in terms of accuracy (0.96), sensitivity (0.95), specificity (0.97), PPV (0.95), and NPV (0.96). When compared to more conventional methods, these findings show that the suggested technique is superior in detecting skin cancer.

In order to better identify skin lesions or cancer, researchers have built an upgraded machine-learning framework^26^. This study segments and categorizes skin diseases and cancers using a machine-learning framework and an optimization method derived from fruit flies. The proposed method provides enhanced classification efficiency with a 98% accuracy rate, 99% specificity, 96% sensitivity, 95% JSI, and 99% DSC.

In^27^, we looked at a framework for multi-class skin disease classification that uses an LSTM, a sophisticated CNN, and MobileNet-V2 to improve the reliability and accuracy of cancer diagnoses. The proposed method leverages CNNs' self-learning discriminative features from unprocessed skin photos and long short-term memory (LSTM) capability to manage multi-class classification problems. The hybrid model showed promise in the experiments for improving the accuracy and efficiency of skin disease classification across a variety of categories.

The authors of^28^ laid up a two-pronged approach to skin lesion categorization. First, during the image pre-processing phase, two distinct techniques were suggested for picture segmentation and feature extraction. Finally, S-MobileNet, a model for a deep convolutional neural network, is being developed with the intention of classifying seven distinct skin lesion types. When it comes to skin lesion picture classification in the HAM10000 dataset, the suggested deep learning-based S-MobileNet model is the way to go, according to experimental results.

In^29^, the authors provide a deep-learning architecture that can identify Melanoma and multi-class skin cancers. Four main phases make up the suggested architecture: pre-processing images, extracting and fusing features, selecting features, and finally, classification. Using the picture luminance data, a new method for enhancing contrast was suggested. The next step is to train two pre-trained deep models, DensNet-201 and DarkNet-53, using transfer learning to modify them with respect to a residual block at the end. The last step in the classification process was to use machine learning classifiers on the features that were chosen. For this experiment, we used two datasets: ISIC2018 and ISIC2019^29,30^. This dataset achieved the highest precision of 85.4%, whereas the other achieved 98.80%. Table 1 presents a comparative analysis of existing skin cancer research using a deep learning model.

**Table 1:** Comparison of existing research for skin cancer analysis.

References	Method	Dataset	Outcome	Hyperparameter optimization
^1^	ResNet50, VGG-16, MobileNet	HAM10000 dataset	Precision 83.97% and 88.25%	Grid search
^2^	DenseNet and Xception	ISIC 2018	Precision 84.37% and 87.17%	Random search
^3^	Shuffle-Net, GoogleNet, MobileNet-V2	ISIC 2020	Precision 89.37%, 90.24% and 89.07%	Hyper-opt
^4^	VGG-19, Res-Net 50, Resnet-152v2	PH-2	Precision 85.08 and 87.62%	Scikit optimize
^5^	VGG-16, VGG-19	ISIC 2020	Precision 83.75% and 84.92%	Optuna
^6^	EfficientNet-V2, VGG-19	Melanoma Skin Cancer Dataset	Precision 86.24% and 87.91%	Search space
^7^	DenseNet, MobileNet-V3	DERMIS Dataset	Precision 91.47% and 89.78%	Grid search
^8^	Efficient-Net, ResNet-50	ISIC 2020	Precision 90.77% and 89.64%	Random search
^9^	Inception-V3, Xception	ISIC-2019 and ISIC-2020	Precision 89.81% and 87.54%	Hyper-opt
^10^	VGG-16 and VGG-19	ISIC 2016	Precision 86.22% and 89.38%	Scikit optimize
^11^	GoogleNet, Efficient-Net	ISIC 2018	Precision 88.74% and 90.51%	Optuna
Proposed Model	Standard U-Net and MobileNet-V3	HAM10000	Precision of 97.84%	Bayesian optimization and grid search

## Materials and methods

### Skin cancer dataset

This research utilizes the online ‘HAM-10000’ skin cancer dataset^33^. The dataset HAM-10000, referred to as 'Human against the machine', with 10,000 images used for training, is a collection of images of skin lesions utilized in skincare research. The dataset comprises visual representations of diverse dermatological disorders, encompassing Melanoma, nevus, and several related afflictions. The provided dataset is used to classify and diagnose skin diseases. As mentioned earlier, deep learning models trained on the dataset can discern different types of skin cancer, including malignant tumours that may exhibit malignant characteristics.

**Figure 2:**
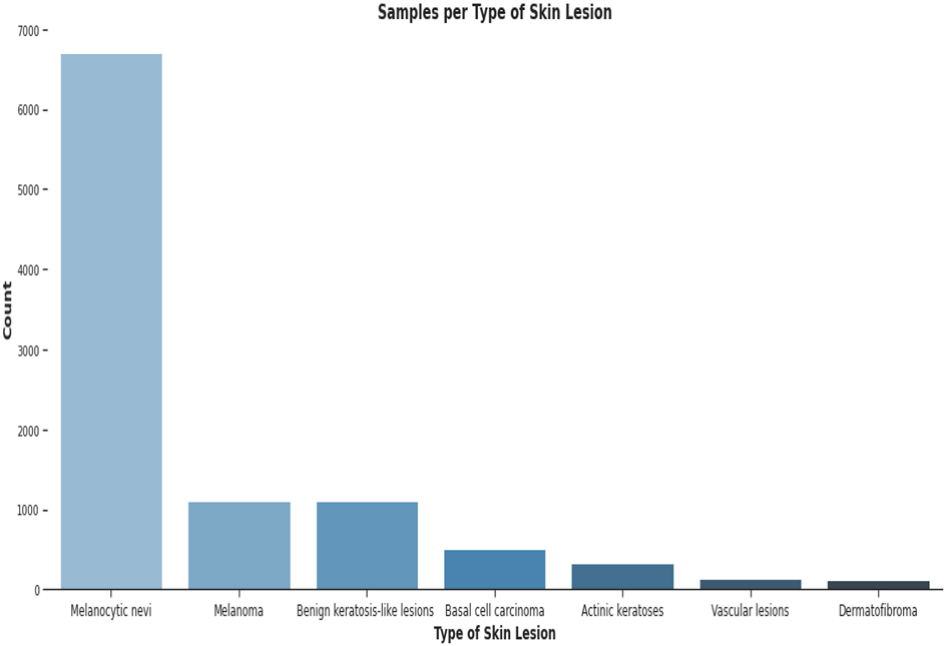
Skin cancer classes and counts.

**Figure 3:**
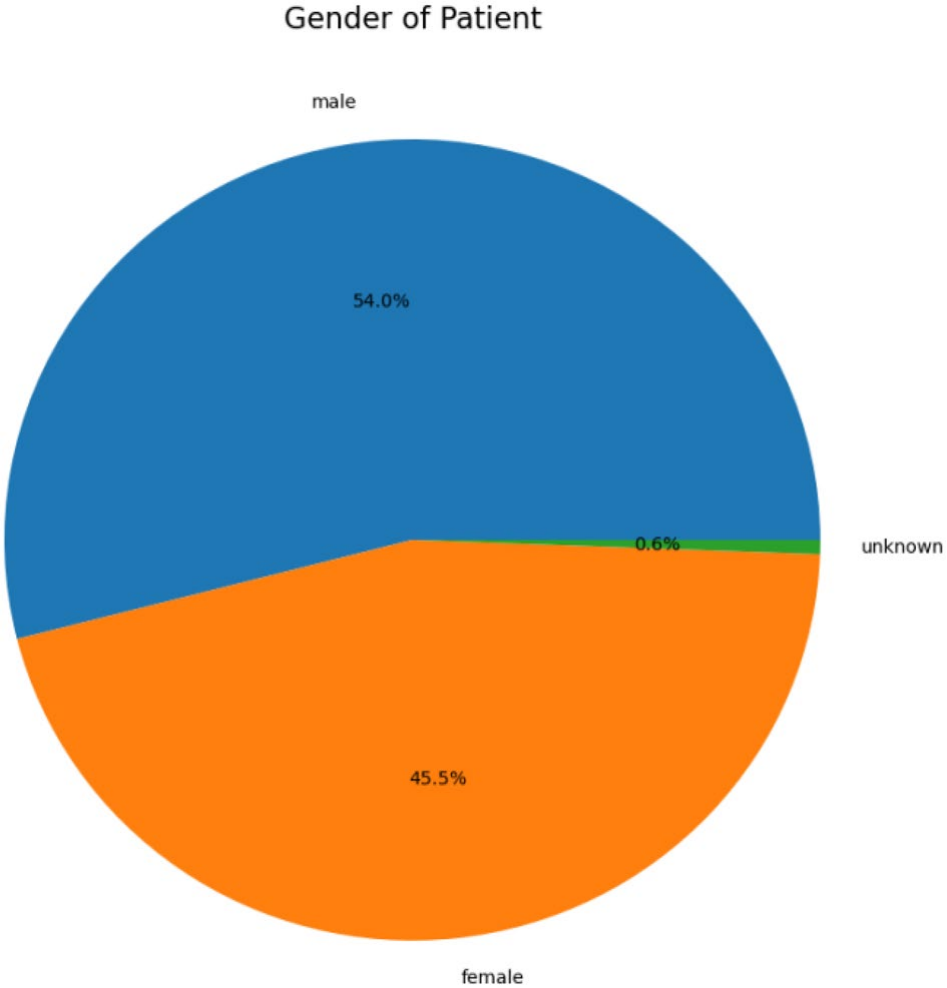
Gender-based skin cancer distribution.

This dataset contains 10,015 dermatoscopic images with size (450*600) pixels. The dataset comprises seven diagnostic Skin cancer categories. The dataset classes include classes from zero to six, names as ‘melanoma’, ‘melanocytic nevus’, ‘basal cell carcinoma’, ‘actinic keratosis’, ‘benign keratosis’, ‘dermatofibroma’ and ‘vascular lesion’. Figure 2 presents the details of skin cancer classes and counts, and Fig. 3 illustrates the gender-wise distribution of skin cancer infection, including a 54% male, 45.5% Female and 0.5% unknown patient distribution in the dataset.

### Data pre-preprocessing

A critical first step in getting a skin cancer dataset ready for training a skin cancer identification framework is data preliminary processing. In addition to promising that the data is in a format appropriate for training, proper data pre-processing can enhance model performance^31^. The skin cancer dataset is pre-processed using the following crucial steps.

#### Data augmentation

**Figure 4:**
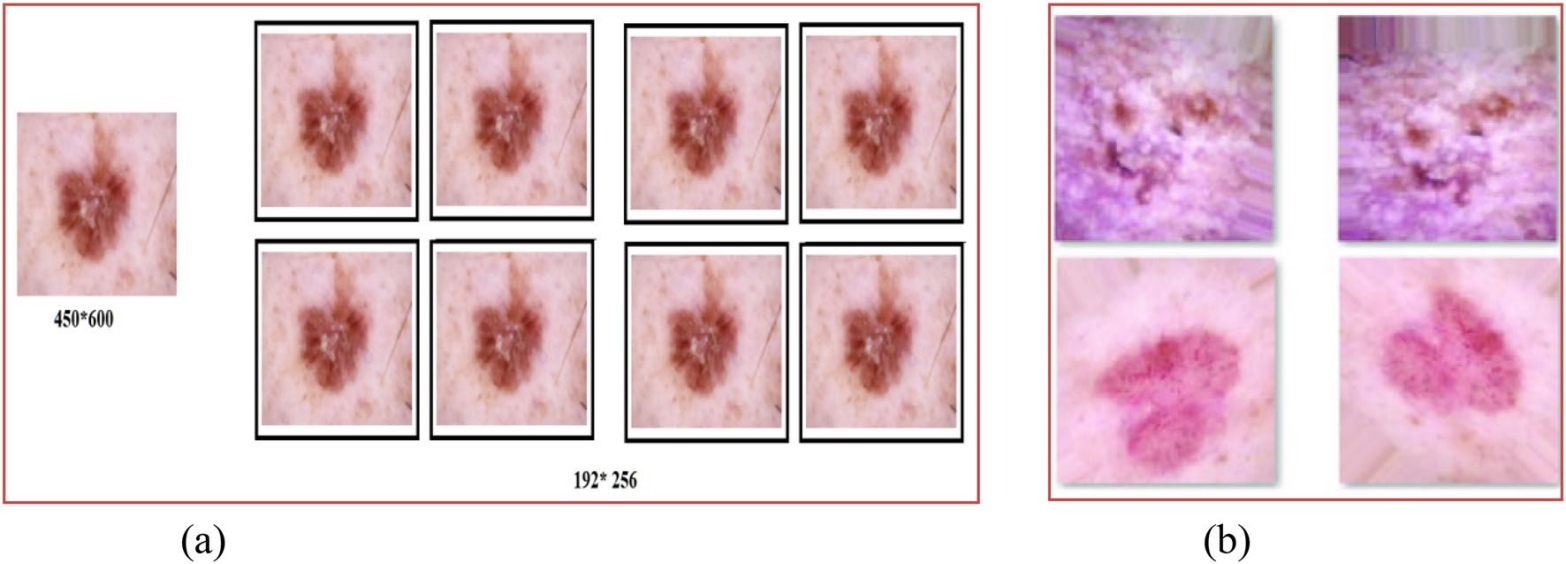
Data augmentation (**a**) image resizing, (**b**) zooming.

Since there are unequal numbers of images in each category, data balancing is used to balance all types before the training procedure. Data augmentation techniques are used for data balancing. In this process, the following ranges are used: (a) rotation 25 degrees; (b) width and height shifting 15%; (c) shearing 15%; (d) employing flipping in both the vertical and horizontal directions; and (5) adjusting brightness within the [0.9: 1.5] range. Data augmentation is also applied to the images during the learning and optimization stages to prevent over-fitting and boost diversity. Table 2 presents the parameters used for the skin cancer dataset. After pre-processing, the images of (450*600) pixels were converted into 192*256 pixels. Figure 4 presents data augmentation results. Table 3 shows the data count after the data pre-processing steps for class 0 to class 6.

**Table 2:** Parameters used for skin cancer data augmentation.

Technique	Value	Augmentation
Zoom range	0.4	Spatial
Horizontal Flip	‘TRUE’	Spatial
Vertical flip	‘TRUE’	Spatial
Shear-range	15	Spatial
Filling mode	“NEAREST” point	Spatial
Rotation-range	25 degree	Spatial
shearing	15%	Spatial
Brightness adjustment	80,150	Pixel
CLAHE	Clip Limit value = 4.0	Pixel

**Table 3:** Data count after pre-processing.

Class type	Label type	Actual data	After Re-sampling
Class-0	‘Melanoma’	6715	7000
Class-1	‘Melanocytic Nevus’	1000	3000
Class-2	‘Basal Cell Carcinoma’	1000	3000
Class-3	‘Actinic Keratosis’	500	1500
Class-4	‘Benign Keratosis’	250	1500
Class-5	‘Dermatofibroma’	300	1500
Class-6	‘Vascular Lesion’	235	1500
Total		10,015	19,000

We use Eq. (1) for horizontal flipping operation, i.e., x-axis, 2 for rotation and 3 for shifting, 4 for shearing, and 5 for zooming. In these equations, h represents the rotating angle, t_x_ represents the amount of shifting in tandem with the x-axis, t_y_ represents the shifting in tandem with the y-axis, sh_x_ represents the shear factor in tandem with the x-axis, sh_y_ represents the shear factor together the y-axis, and C_x_ represents the zoom factor together the x-axis, and C_y_ represents the zoom factor in tandem the y-axis^32^. Here, FM is the flipping Matrix, RM is the rotation Matrix, SM is the shifting Matrix, SheM is the shearing Matrix, and ZM is the zooming Matrix.

1 $$FM=\left[\begin{array}{ccc}1& 0& 0\\ 0& cos\theta & -sin\theta \\ 0& sin\theta & cos\theta \end{array}\right]$$

2 $$RM=\left[\begin{array}{ccc}cos\theta & sin\theta & 0\\ -sin\theta & cos\theta & 0\\ 0& sin\theta & 1\end{array}\right]$$

3 $$SM=\left[\begin{array}{ccc}1& 0& 0\\ 0& 1& 0\\ {t}_{x}& {t}_{y}& 1\end{array}\right]$$

4 $$SheM=\left[\begin{array}{ccc}1& s{h}_{y}& 0\\ s{h}_{x}& 1& 0\\ 0& 0& 1\end{array}\right]$$

5 $$ZM=\left[\begin{array}{ccc}{c}_{x}& 0& 0\\ 0& {c}_{y}& 0\\ 0& 0& 1\end{array}\right]$$

### Proposed model architecture

**Figure 5:**
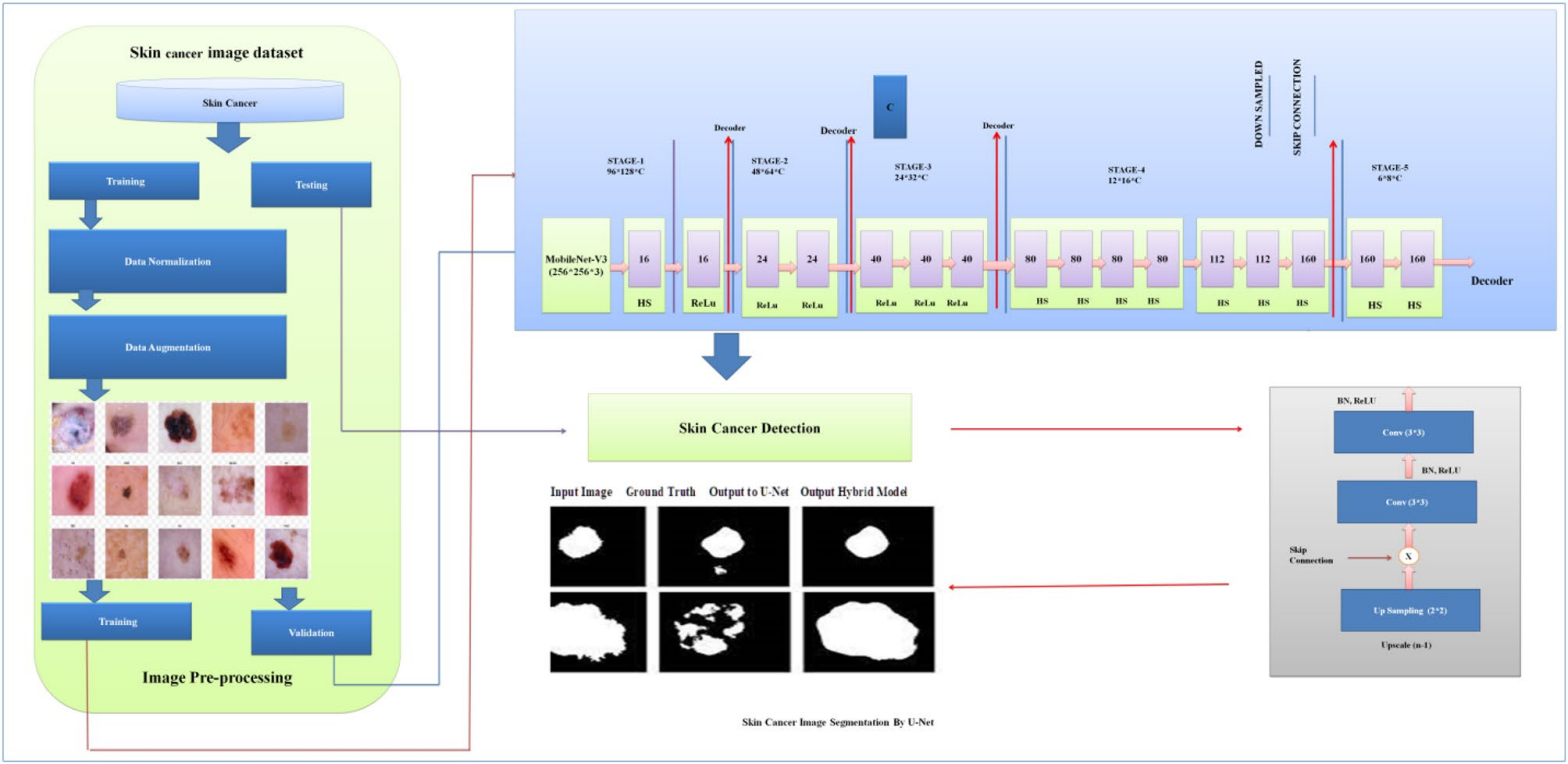
Architecture of proposed hybrid model.

The proposed model is based on Standard U-Net and Improved MobileNet-V3 with optimization techniques, i.e., the Bayesian optimization method. Standard U-Net performs semantic segmentation activities in the proposed model, and MobileNet-V3 is used as a Feature Extractor. We also utilize a Bayesian optimization method for hyperparameter optimization. This method can identify the most promising hyperparameters by prioritizing those that have shown favourable outcomes in previous results. Figure 5 shows the architecture of the proposed hybrid model.

#### Improved MobileNet-V3

**Figure 6:**
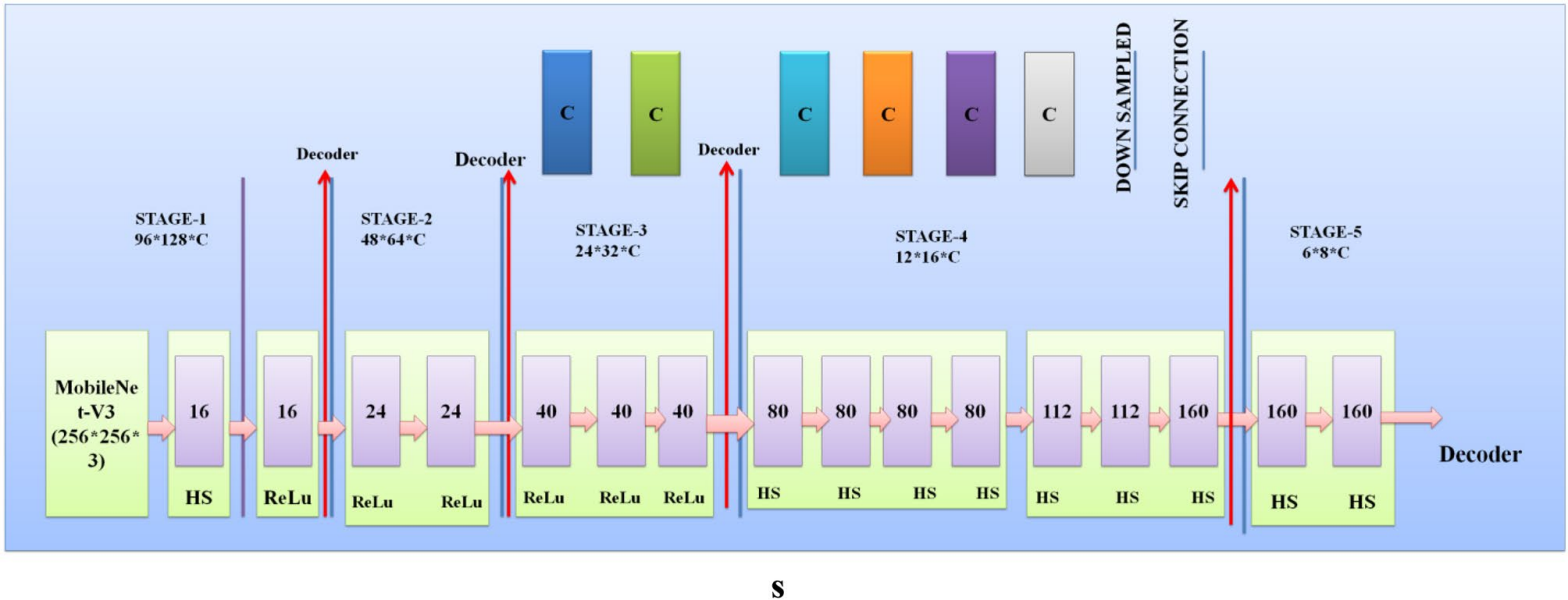
Architecture of proposed MobileNet-V3.

In the proposed hybrid model, the existing MobileNet-V3 model is improved. The Improved MobileNet-V3 worked as an encoder in the proposed skin cancer prediction and segmentation model. The steps below enhanced the MobileNet-V3 structure for skin cancer recognition and segmentation. Figure 6 presents the architecture of the proposed MobileNet-V3^31^.

Architectural level Improvements: The following fundamental changes were performed at the existing MobileNet-V3 architecture^32^.

- *Deeper Model* A deeper MobileNet-V3 model's depth (layer counts) is determined by the model's structural preferences. MobileNet-V3 is available in two predetermined sizes: "large" or "small," with "large" featuring more layers and greater capacity than "small." The "large" variant of MobileNet-V3 generally includes 157 layers. Depthwise separable convolution layers and flipped residuals using growing and squeeze-and-excitation (SE) segments are formed.
- *Wider Model* MobileNet-V3 predominantly employs the flipped residual block and SE block. A depthwise convolution component is subsequently followed by a point-wise convolution layer to form the inverted residual block. The point-wise convolution changes the width (number of channels). Adjust the point-wise convolution layer output channels to widen a layer. This is typically accomplished by multiplying the total amount of channels through a scaling factor. Modulating channel-wise dependencies is done with the SE block. Although it cannot naturally regulate layer width, it can be utilized on layers to enhance model performance and adjust to particular tasks. Fully connected layers (FCL), Global average pooling (GAP), and element-wise scaling (EMS) comprise the SE block. The number of channels present in the map of input features is used as a criterion to establish the size of the SE block. MobileNet-V3 allows you to modify the range of channels within the starting convolutional layer with the last classification layer to adjust the total width of the resulting model for skin cancer analysis.
- *Attention Method* We substituted the crossed-entropy loss function of the Mobilenet-v3 mathematical framework with a bias loss function that enhanced accuracy. The model's SE component was replaced with the adequate channel attention (ECA) component to achieve parameter reduction. Integrating cross-layer connections among Mobile modules has been proposed to leverage synthetic features effectively. The dilated convolutions were incorporated into the model to enhance the receptive field.

##### Regularization method

The following Regulization techniques were applied to improve the MobileNet-V3 model^32^.

- *Dropout* Implement dropout layers inside the MobileNet-V3 structure to prevent overfitting throughout training. Dropout arbitrarily eliminates a portion of the neurons throughout each forward pass to improve the model's generalization ability.
- *L2 regularization's weight decay* Applying a weight decay component to the model's loss function for the L2 regularization's weight decay step. Weight decay promotes the reduction of model weights, preventing unnecessary complexity and overfitting.

##### Normalization method

The following Normalization techniques were applied for improvements in the MobileNet-V3 model.

- *Batch Normalization* To stabilize training, employ batch normalization layers to normalize the activations throughout each mini-batch. Batch normalization can boost the model convergence process and boost training.
- *Layer Normalization* We have applied a layer normalization method that helps

#### Standard U-Net architecture

The Standard U-Net architecture is a convolutional neural network specifically developed for semantic segmentation tasks. The algorithm demonstrates exceptional proficiency in partitioning regions of interest within medical images, rendering it highly suitable for the analysis of skin cancer. The Standard U-Net architecture is distinguished by incorporating skip connections and embedded skip procedures, which efficiently capture multi-scale context-relevant data^33^.

**Figure 7:**
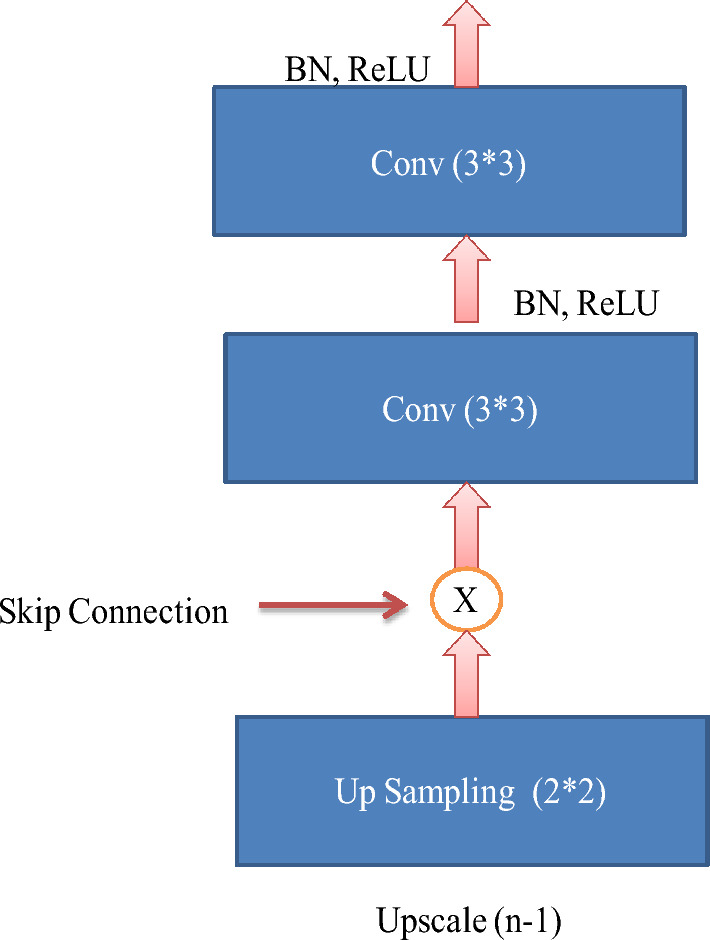
Standard U-Net architecture.

Four Standard U-Net patterns, identical to U-Net, are used to complete the segmentation assignment. Batch normalization and GeLU for the hidden activation mechanism are utilized in the additional configuration, while the architecture described in^10^ is used in the initial setup. Batch normalization (BN) mainly creates batches of a specific size. Along with batch normalization and the GeLU hidden activation function, the third and fourth patterns use VGG-19 and DenseNet-201 as their backbone. In the case of configuration four, deep supervision is not enabled. Figure 7 presents the standard U-Net architecture.

#### Bayesian optimization method

Bayesian optimization is an approach that employs probabilistic algorithms to search accurately for the most practical combination of hyperparameters to support a model built using deep learning. It functions by analyzing an ambiguous objective function within this instance, which evaluates the model's effectiveness as a Gaussian procedure, enabling it to make well-informed choices about where to start sampling the target function subsequently. The following describes the operation of Bayesian optimization in the hyperparameter optimization for skin cancer^6^.

- *Step 1: Initial Random Sampling* To create an initial substitute framework for the objective function, Bayesian optimization usually begins with a few random specimens taken through the hyperparameter space. The function's behaviour can be partially understood because of these arbitrary specimens.
- *Step 2: Surrogate Model* The algorithm generates a probabilistic substitute model using the initial specimens, generally a Gaussian procedure (GP). The GP prototypes the objective function's allocation and predicts the function's behaviour throughout the hyperparameter area. This substitute model encompasses average forecasting and an uncertainty determination (variance) at every point within the hyperparameter space.
- *Step 3: Acquisition Function* Bayesian optimization utilizes an acquisition function for identifying the subsequent objective function specimen location. Typical acquisition functions consist of Probability of Improvement (PI), Expected Improvement (EI), and Upper Confidence Bound (UCB). These features balance examinations (sample collection in spaces of uncertainty) and exploitation (sampling in spots that are likely to produce more effective results).
- *Step 4: Next Sample Selection* The acquisition function directs the selection of the subsequent collection of hyperparameters to be assessed. The method chooses hyperparameter values that maximize the acquisition function, indicating promising regions within the hyperparameter space for enhancing the objective function.
- *Step 5: Evaluate Objective Function* The chosen hyperparameters are utilized for training and evaluating a deep learning framework via the skin cancer dataset. The objective function (including model precision and loss) gets calculated according to the model's efficiency.
- *Step 6: Update Surrogate* Model: After assessing the objective function based on the chosen hyperparameter, the procedure upgrades the substitute model (GP) along with the newly acquired data point, including the evaluation results, to minimize uncertainty within the substitute model.
- *Step 7: Iteration* Steps 3 through 6 are carried out for an established amount of iterations or until convergence is achieved. The method improves the substitute model iteratively and chooses a hyperparameter to enhance the model's accuracy.
- *Step 8: Final Result* The Bayesian optimization method produces the hyperparameters, which generate the best objective function importance according to the model's behaviour.

#### Transfer learning

ImageNet is used as a pre-training dataset for the MobileNet-V3 model, and then the Ham-10000 skin cancer dataset is used to fine-tune the model's parameters. Transfer learning enables the predictive algorithm to utilize features acquired within a broader context before becoming proficient in the skin cancer recognition assignment^34^.

### Pseudo code for the proposed model

Algorithm 1 presents the steps for skin cancer analysis with the hybrid U-Net and MobileNet-V3 models^35^.

**Algorithm 1:**
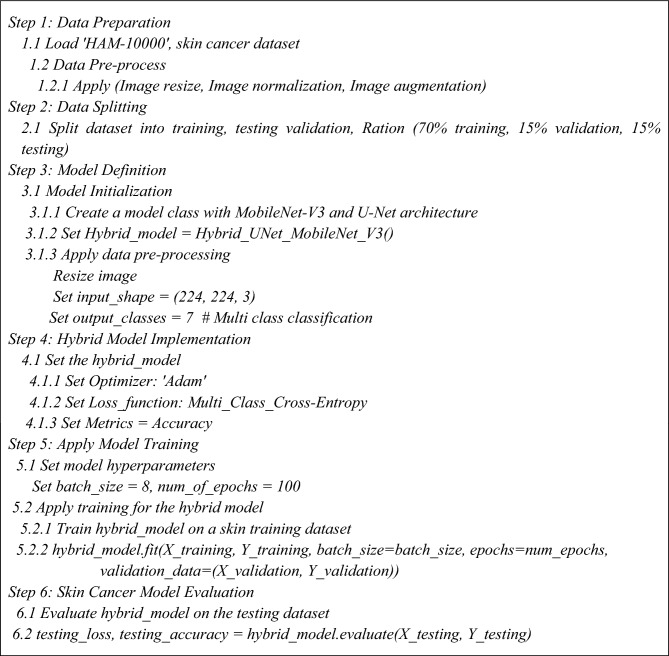
Skin Cancer analysis with Hybrid U-Net and MobileNet-V3.

### Performance measuring parameters

We use parameters like accuracy, precision, F-measure, the area under the curve (AUC), and confusion metrics^6,31–33^ to determine how to determine the present model and the one we have suggested works. The components of the confusion matrix, including TP (true positive), TN (true negative), FP (false positive), and FN (false negative), have been utilized in its construction. The subsequent passage comprehensively explains the previously referenced statistics^34^.

- *Precision (Prc)* This illustrates the proportion of individuals who test positive correctly (true positives) and incorrectly (false positives) concerning the total number of positive individuals as described in Eq. (6).
  6 $$Prc=[\frac{TP}{(TP+FP)} ]$$
- *Recall (RC) or Sensitivity* The calculation of recall, also known as true positive rate^35^, involves dividing the number of true positives by the total number of instances that ought to be considered anticipated as positive, as described in Eq. (7).
  7 $$RC=[\frac{TP}{\left(TP+FN\right)} ]$$
- *F-Measure (FMs)* The F-score, often referred to as the 'F1 score' or 'F-measure'^36^, serves as an empirical metric for assessing the effectiveness of a deep learning model. The integration of recall and precision results in an integrated score, as described in Eq. (8).
  8 $$FMs=\{2*[\frac{ Prc \times RC}{ Prc+RC}] \}$$
- *Accuracy (ACR) *The evaluation of classification models often involves the consideration of various metrics, with accuracy being one of them^37^. In a more formal context, accuracy can be defined as the proportion of correct predictions our model makes, as described in Eq. (9).
  9 $$ACR=\{\frac{(TP+TN)}{[(TP+FN)+(TP+FP)]} \}$$
- *Area Under the Curve (AUC)* AUC refers to measuring the extent or size of two areas under a curve, which refers to the region bounded by the curve and the coordinate axes^33,38^. To determine this area, one can divide it into infinitesimally small rectangles and sum their areas. By incorporating the limit of this summation, as the rectangles become infinitely small, the total area under the curve can be calculated.

### Ethical approval and consent to participate

No ethical approval is required, and the authors consent to participate in the paper.

## Experimental results and evaluation

This section covers the simulation results of the existing and proposed model.

### Experimental setup

The proposed and existing techniques, i.e., MobileNet, Resnet-152v2, VGG-16, MobileNet-V2, and VGG-19, were implemented using software and hardware components. Table 4 presents the parameters used in experimental analysis for proposed and existing models. Table 5 shows the Configuration parameters of Standard U-Net architecture used in a proposed hybrid model for image segmentation^39–43^.

**Table 4:** Experimental Parameters for Proposed Model.

Parameters	Details
Batch size	8
Data augmentation strategies	Flipping, zooming, translations and rotations,
Normalization	0, 1
Regularization	L2 regularization (Weight decay)
Optimizer	‘Adam optimizer’
Dropout rate	0.1
Epochs	100
Image input size	(224 × 224)
Hyperparameter optimization	Bayesian optimization
Transfer learning	MobileNet-V3
Loss function	Multi-class categorical cross-entropy function
Learning rate	0.2
Growth rate	24
Split ratio	Training: testing: validation 70: 15: 15
Shuffling in database	“YES”
Brightness range	[0.2, 2.25]
Rotation	0 to 15 Degrees

**Table 5:** Configuration parameters of Standard U-Net.

Model	Activation function	Stack down	Stride	Backbone	Stack up	Batch normalization	Pooling	Pooling
Standard U-Net	ReLu	2	1	None	2	‘TRUE’	‘TRUE’	‘FALSE’

### Simulation results and discussion

The HAM-10000 Skin cancer dataset serves as the training ground for the proposed model and several existing deep learning models, i.e., MobileNet, Resnet-152v2, VGG-16, MobileNet-V2, and VGG-19. The dataset was divided into training, testing and validation with a ratio of 70:15:15. The experimental results and discussion are as follows.

Table 6 presents the experimental results of the testing dataset for 100 epochs for existing and proposed models. The combined utilization of the U-Net and MobileNet-V3 models yielded exceptional results in classifying skin cancer diseases.

**Table 6:** Experimental results of Testing for 100 epochs.

Model	Precision %	Recall %	F-1 Score %	Accuracy %	ROC-AUC %
MobileNet^1^	91.25	89.18	88.34	92.45	96.25
Resnet-152v2^2^	90.34	88.57	87.65	92.57	95.32
VGG-16^5^	90.78	89.27	88.95	92.89	94.21
MobileNet-V2^3^	92.36	90.78	89.36	94.47	91.78
VGG-19^10^	93.65	91.47	90.65	94.89	93.54
Proposed model	97.84	95.27	97.32	98.86	98.45

Table 7 presents the experimental results of the validation dataset for 100 epochs for existing and proposed models. The proposed model demonstrated validation results with an accuracy of 98.03%, wherein the precision, recall, and F1-score all surpassed 94%.

**Table 7:** Experimental results for validation (100 epochs).

Model	Precision %	Recall %	F-1 Score %	Accuracy %	ROC-AUC %
MobileNet^1^	90.18	89.03	87.89	92.02	95.89
Resnet-152v2^2^	90.03	88.14	87.02	92.07	95.03
VGG-16^5^	90.07	89.02	88.03	92.05	94.01
MobileNet-V2^3^	91.98	90.01	89.01	94.03	91.07
VGG-19^10^	93.01	90.09	88.04	94.08	93.02
Proposed model	97.32	94.07	97.03	98.03	97.09

### Visualization of simulation results

**Figure 8:**
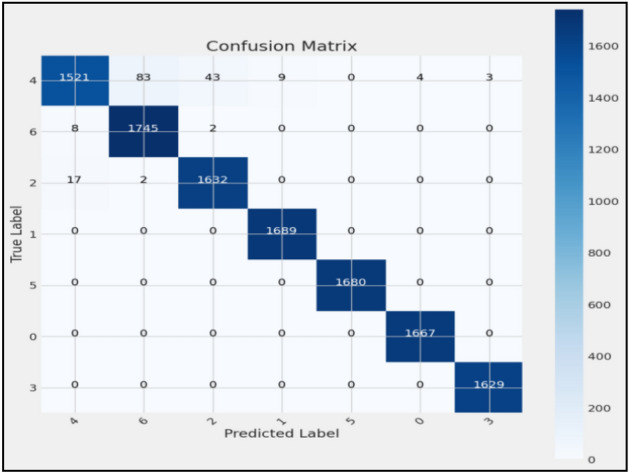
Confusion matrix of proposed model.

**Figure 9:**
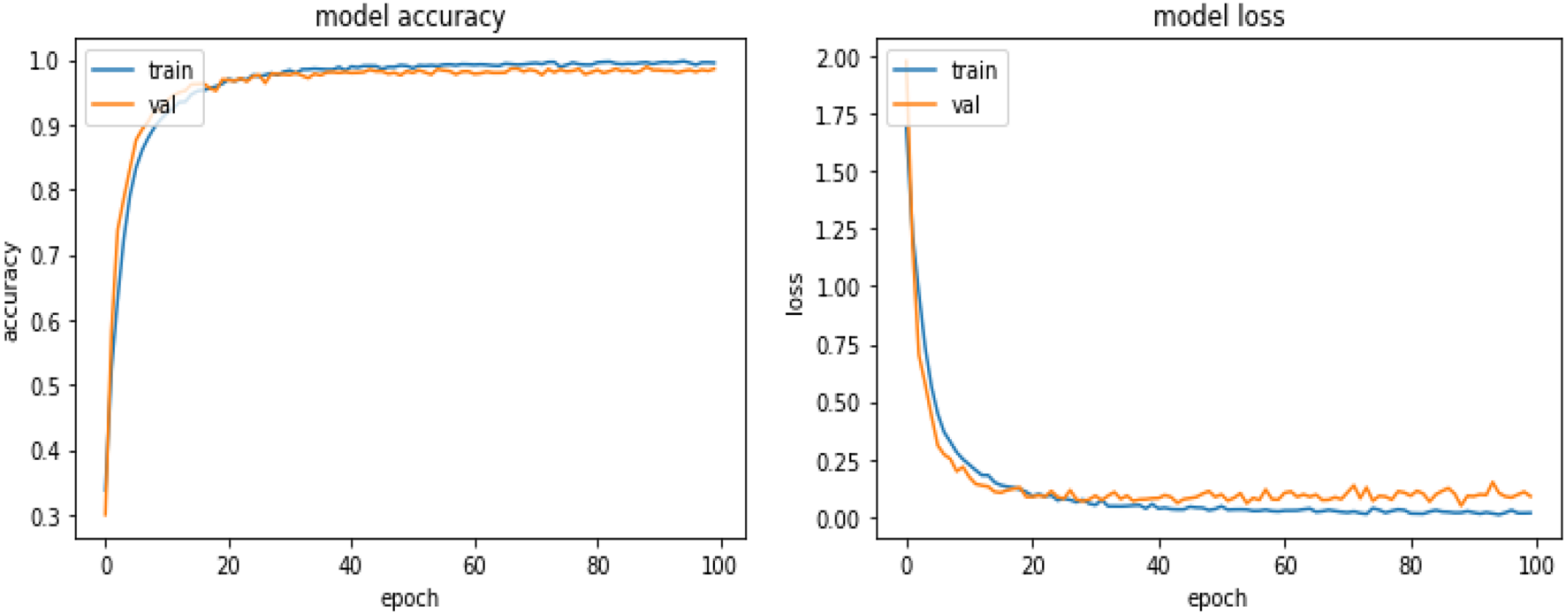
Model accuracy and loss of proposed model.

**Figure 10:**
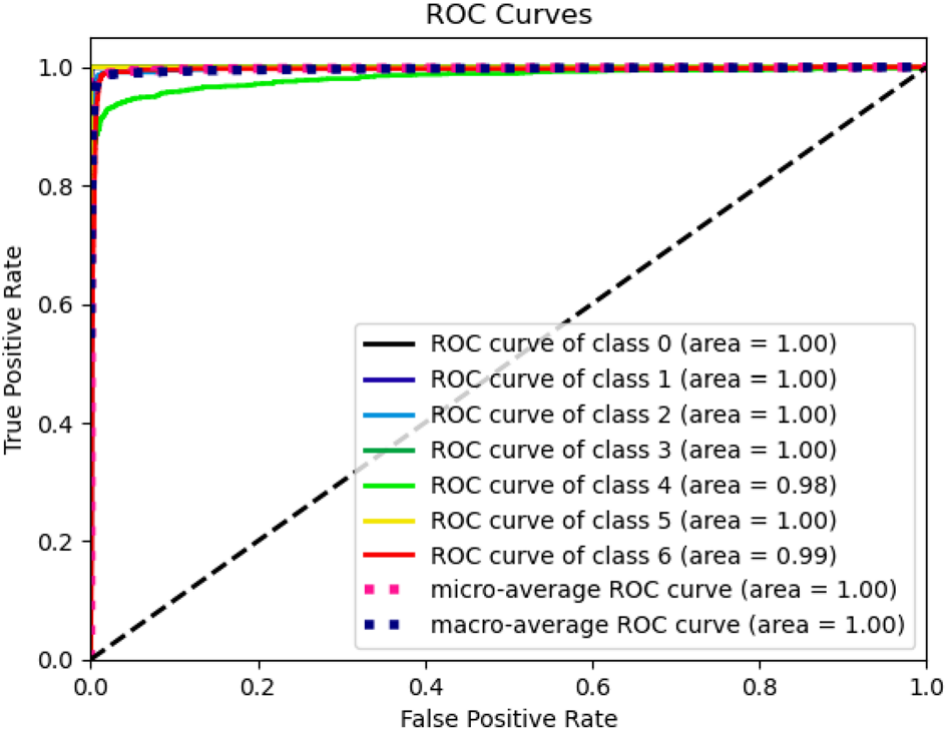
ROC curve of proposed model.

**Figure 11:**
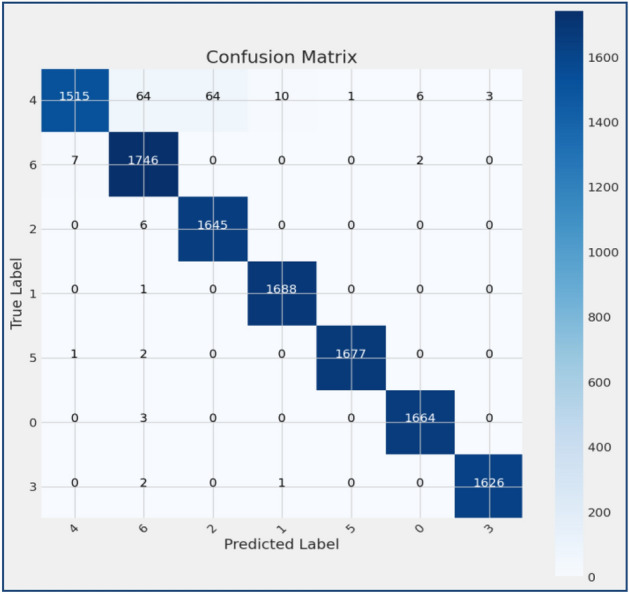
Confusion matrix of existing MobileNet model.

**Figure 12:**
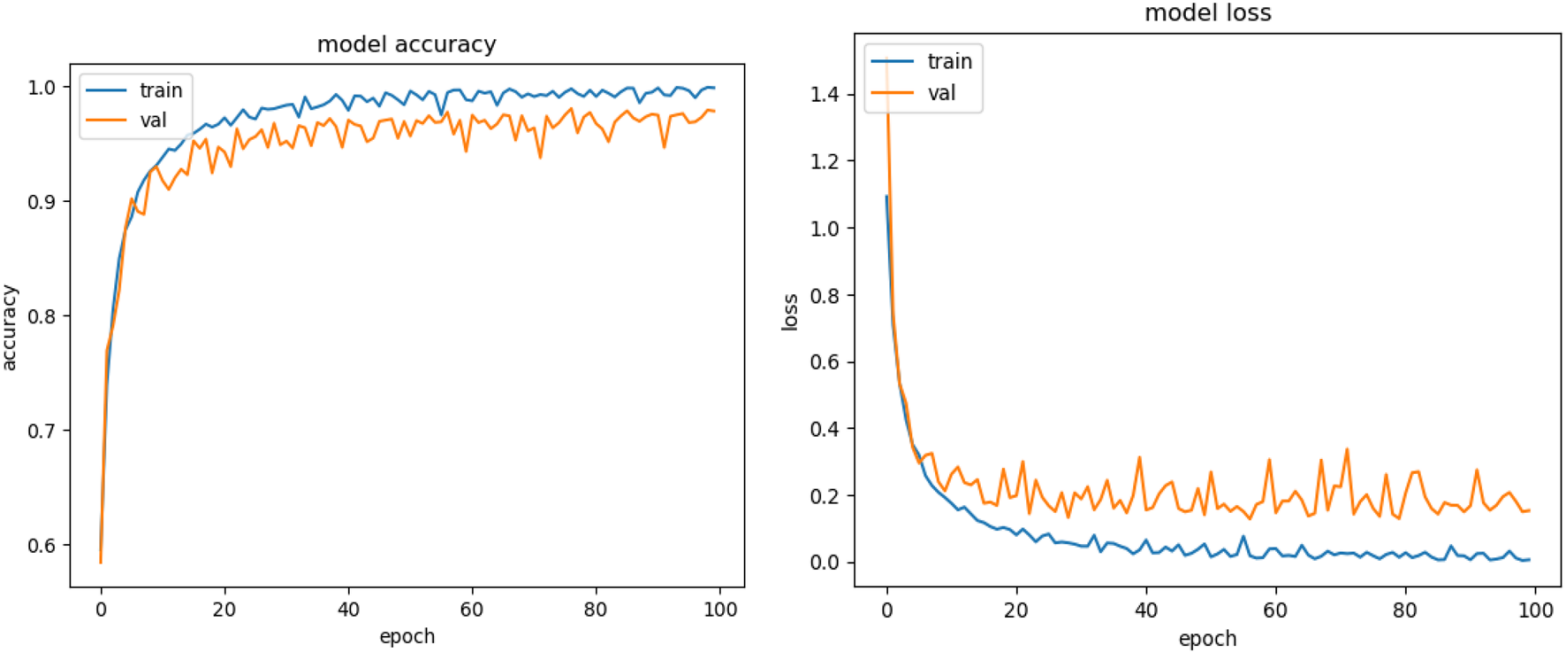
Model accuracy and loss of Existing MobileNet model.

**Figure 13:**
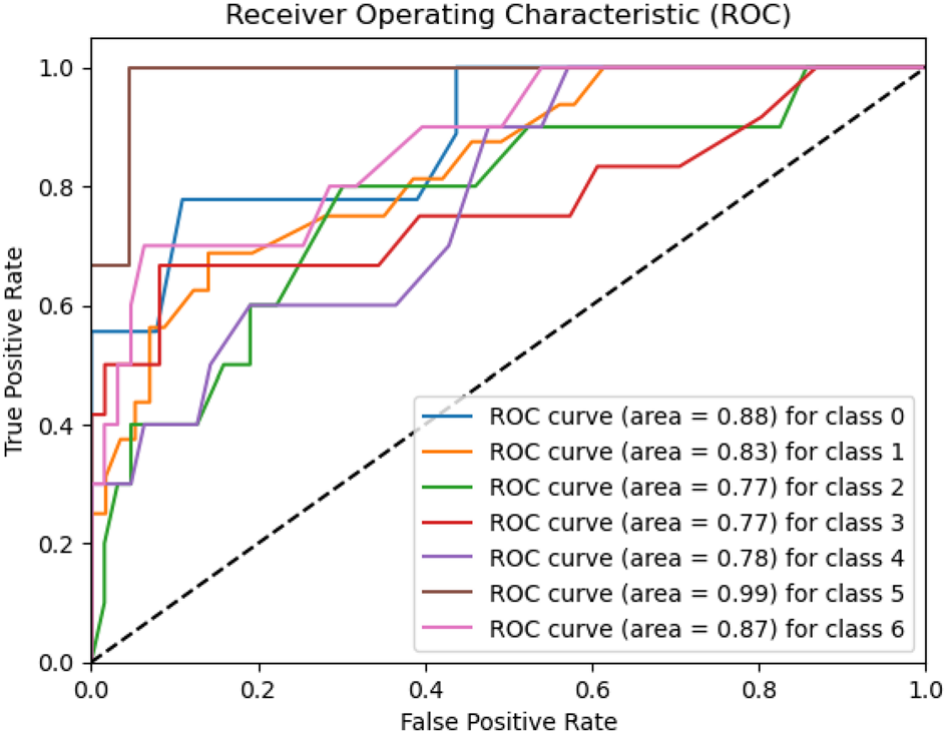
ROC curve of existing MobileNet model.

Figures 8, 9, 10 present a visualization of simulation results for the proposed model on the “HAM-10000” skin cancer dataset. Figure 8 presents the Confusion Matrix of the Proposed Model, Fig. 9 illustrates the Model Accuracy and Loss of the Proposed Model, and Fig. 10 shows an ROC Curve of the Proposed Model. Figures 11, 12,13 present a visualization of simulation results for the MobileNet Model on the “HAM-10000” skin cancer dataset. Figure 11 presents the Confusion Matrix of the MobileNet Model, Fig. 12 illustrates the Model Accuracy and Loss of the MobileNet Model, and Fig. 13 shows an ROC Curve of the MobileNet Model.

**Figure 14:**
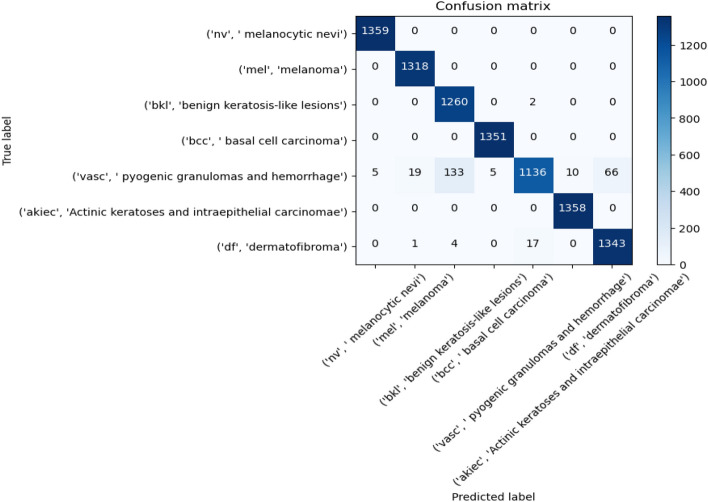
Confusion matrix existing MobileNet-V2 model.

**Figure 15:**
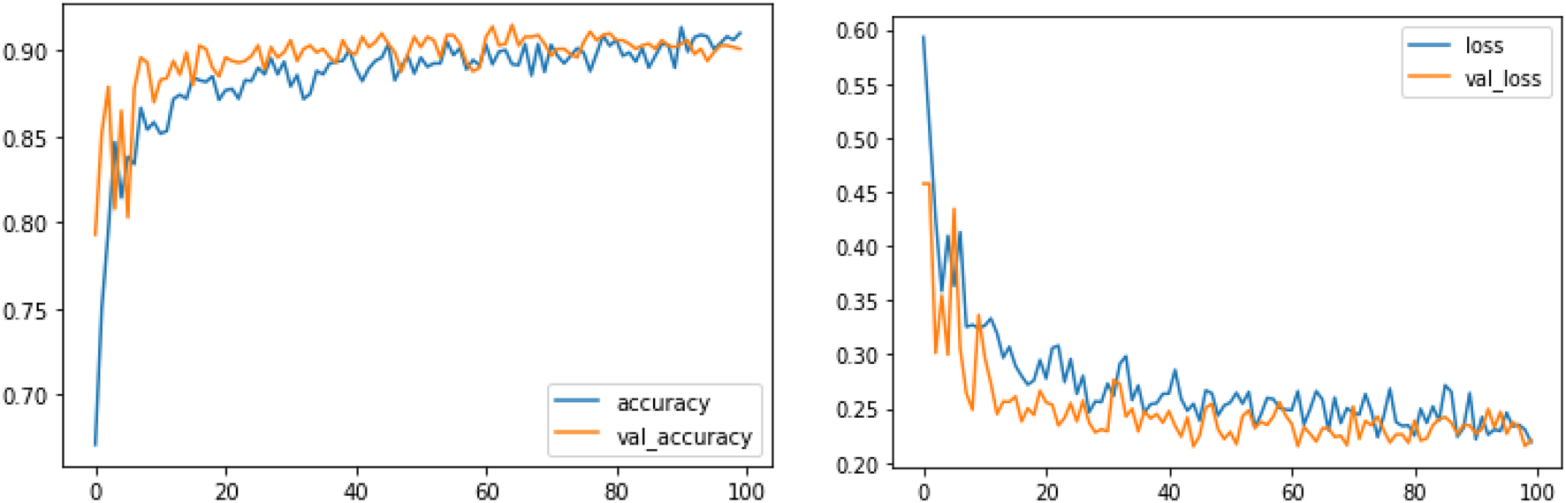
Model training, validation accuracy and loss existing MobileNet-V2 model.

**Figure 16:**
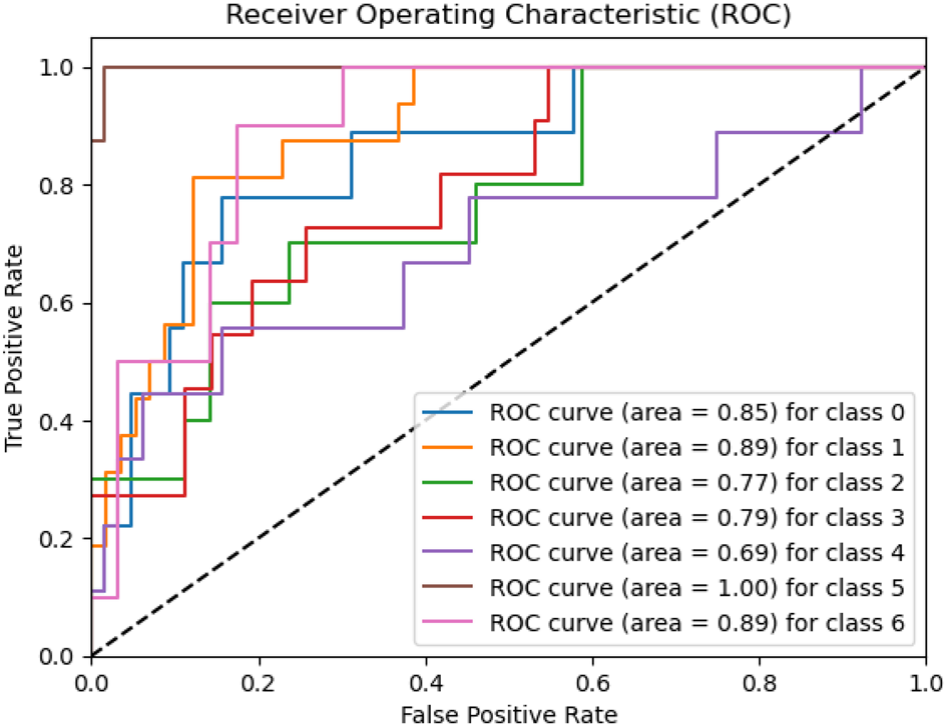
ROC curve of existing MobileNet-V2 model.

Figures 14, 15, 16 present a visualization of simulation results for the MobileNet-V2 Model on the “HAM-10000” skin cancer dataset. Figure 14 presents the Confusion Matrix of the MobileNet-V2 Model, Fig. 15 shows the Model Accuracy and Loss of the MobileNet-V2 Model, and Fig. 16 illustrates an ROC Curve of the MobileNet-V2 Model.

**Figure 17:**
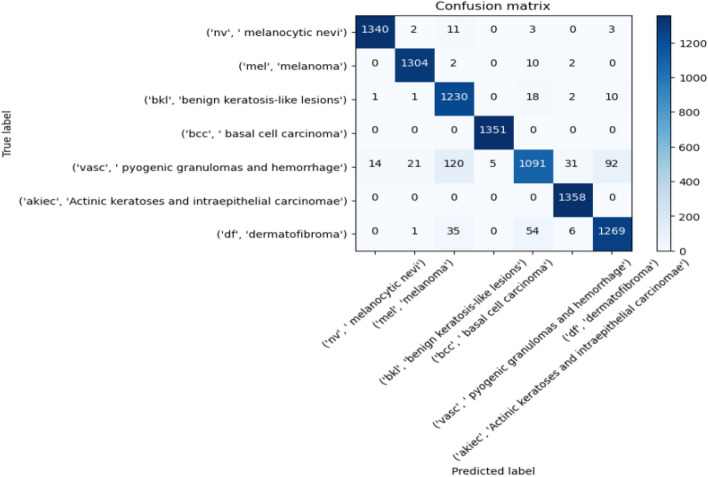
Confusion matrix of existing VGG-16 model.

**Figure 18:**
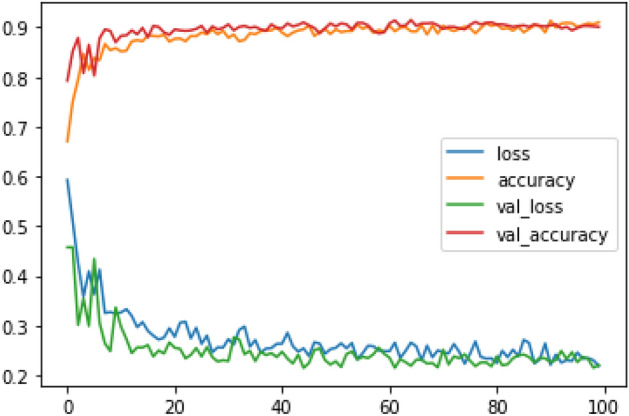
Model training, validation accuracy and loss existing VGG-16 model.

**Figure 19:**
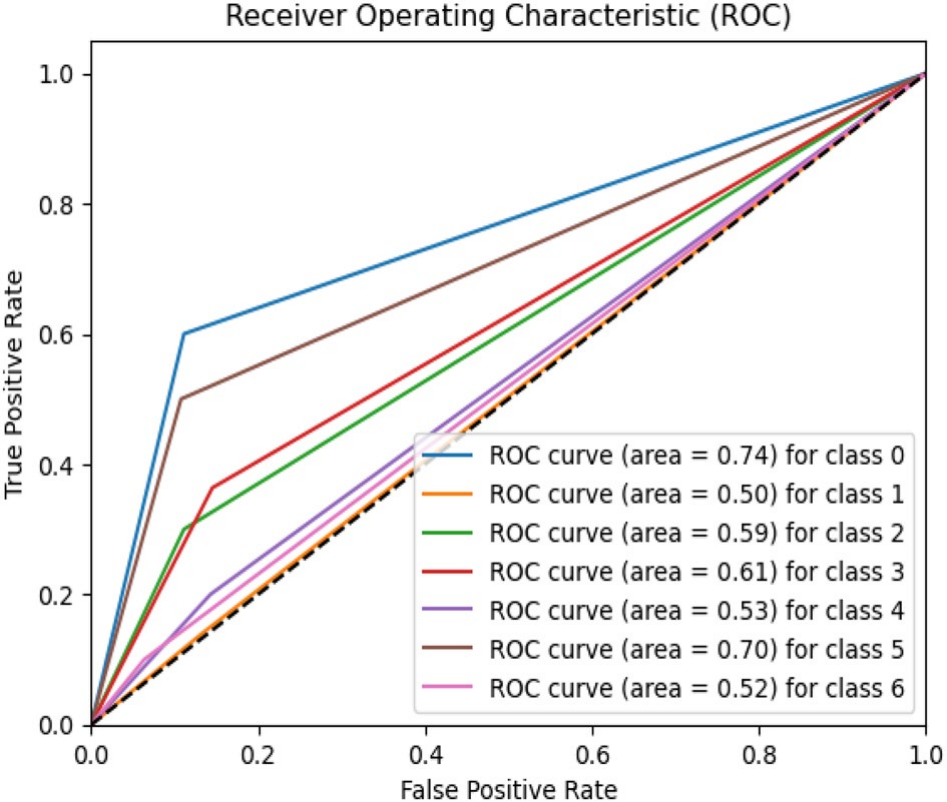
ROC curve of existing VGG-16 model.

Figures 17, 18, 19 present a visualization of simulation results for the VGG-16 Model on the “HAM-10000” skin cancer dataset. Figure 17 presents the Confusion Matrix of the VGG-16 Model; Fig. 18 illustrates the Model Accuracy and Loss of the VGG-16; Fig. 19 shows an ROC Curve of the VGG-16 Model.

**Figure 20:**
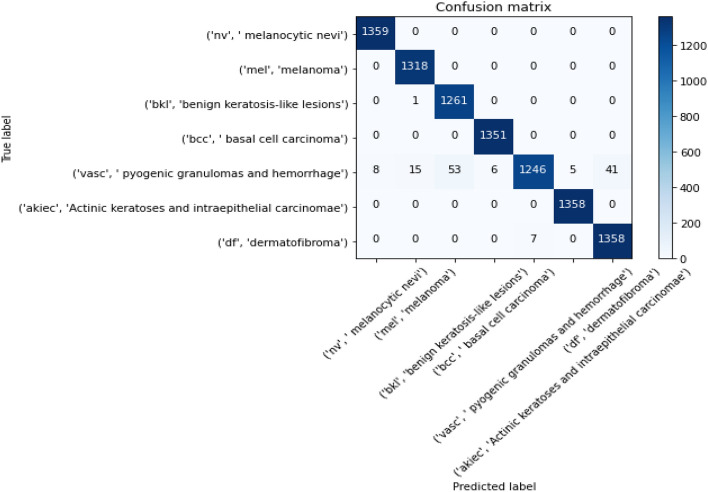
Confusion matrix of existing VGG-19 model.

**Figure 21:**
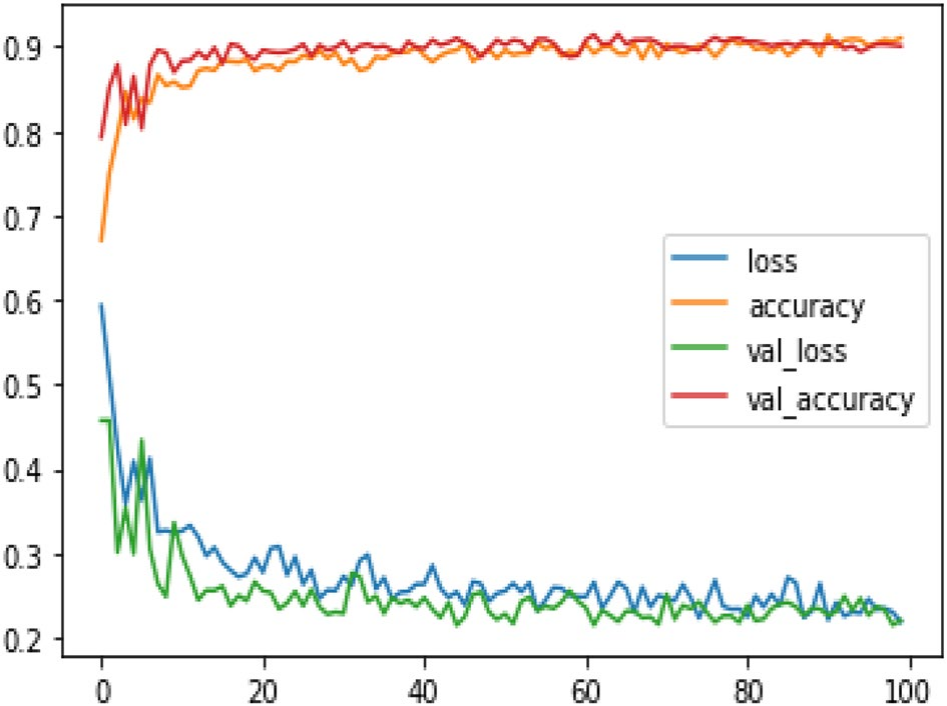
Model training accuracy and validation of existing VGG-19 model.

**Figure 22:**
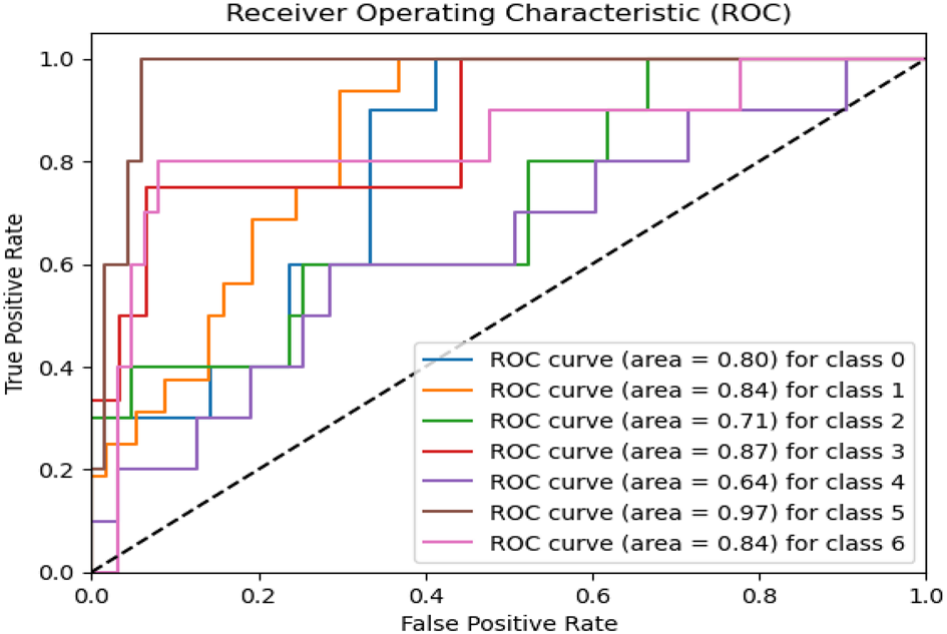
ROC curve of existing VGG-19 model.

**Figure 23:**
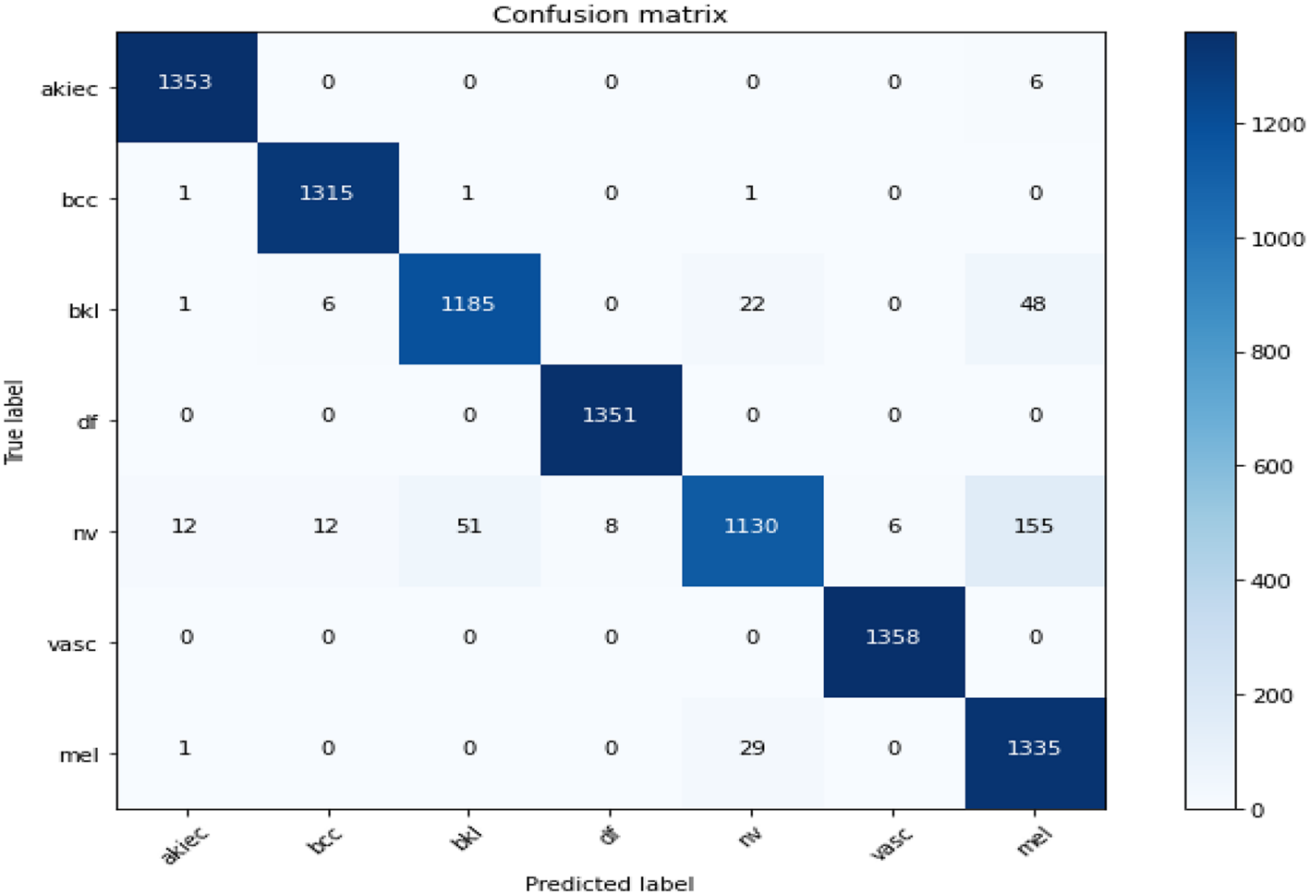
Confusion matrix of resnet-152v2.

**Figure 24:**
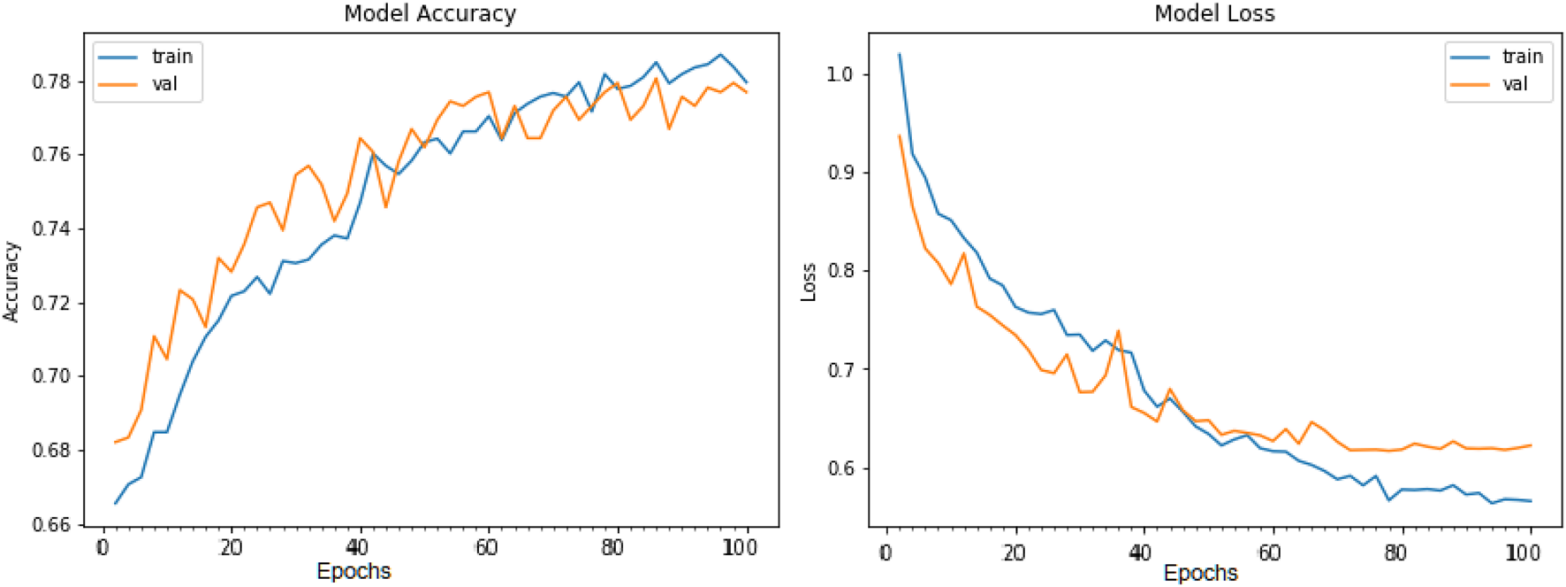
Training and validation accuracy, loss results of resnet-152v2.

**Figure 25:**
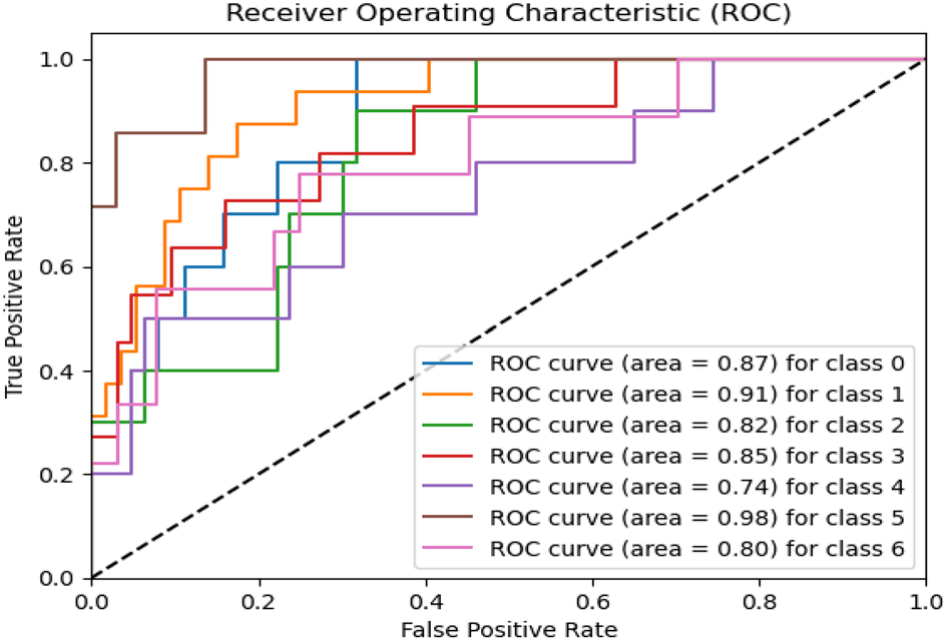
ROC-curve of resnet-152v2.

Figures 20, 21, 22 present a visualization of simulation results for the VGG-19 Model on the “HAM-10000” skin cancer dataset. Figure 20 presents the Confusion Matrix of the VGG-19 Model; Fig. 21 illustrates the Model Accuracy and Loss of the VGG-19; Fig. 22 shows an ROC Curve of the VGG-19 Model. Figures 23, 24, 25 present a visualization of simulation results for the Resnet-152v2Model on the “HAM-10000” skin cancer dataset. Figure 23 presents the Confusion Matrix of the Resnet-152v2 Model; Fig. 24 illustrates the Model accuracy and loss of the Resnet-152v2; Fig. 25 shows an ROC Curve of the Resnet-152v2 Model. Based on the analysis of the results, it’s clearly proven that the proposed model has better simulation outcomes than existing models.

### Results and discussion

The HAM-10000 Skin cancer dataset is utilized for the proposed model and several existing deep learning models, i.e., MobileNet, Resnet-152v2, VGG-16, MobileNet-V2, and VGG-19. Table 6 presents the experimental results of the testing dataset for 100 epochs for existing and proposed models. The combined utilization of the U-Net and MobileNet-V3 models yielded exceptional results in classifying skin cancer diseases. The model demonstrated a set of validation results with an accuracy of 98.86%, wherein the precision, recall, and F1-score all surpassed 95%. The model's capability to distinguish between cancerous and non-cancerous tumours is demonstrated by the ROC-AUC score of 98.45%, indicating excellent performance. The model consistently demonstrated a strong performance on the test set, achieving an accuracy rate of 98.45%.

The evaluation metrics, precision, accuracy, recall, ROC-AUC and F1-score, exhibited remarkable performance on the testing dataset, surpassing 95% for each metric. The ROC-AUC result of 98.45% reaffirms the model's robustness compared to the proposed model, existing MobileNet achieved, reaching 89% for each performance metric. Resnet-152V2 was achieved, surpassing 88% for each performance metric; VGG-16 was achieved, exceeding 89% for each performance metric. MobileNet-V2 achieved, exceeding 90% for each performance metric, and VGG-19 achieved, surpassing 90.50% for each performance metric. These results indicate that the proposed model achieved outstanding results over existing deep learning models.

Table 7 presents the experimental results of the validation dataset for 100 epochs for existing and proposed models. The proposed model demonstrated validation results with an accuracy of 98.03%, wherein the precision, recall, and F1-score all surpassed 94%. The model's capability to distinguish between cancerous and non-cancerous tumours is demonstrated by the ROC-AUC score of 97.09%, indicating excellent performance. The evaluation results show precision, accuracy, recall, ROC-AUC, and F1-score, exhibiting remarkable performance on the validation dataset, surpassing 94% for each metric. The ROC-AUC result of 97.09% reaffirms the model's robustness. As compared to the proposed model, the existing model, i.e., MobileNet achieved, surpassing 87% for each performance metric, Resnet-152V2 achieved, surpassing 87.02% for each performance metric VGG-16 achieved, reaching 88% for each performance metric, MobileNet-V2 achieved, exceeding 89% for each performance metric, and VGG-19 achieved, surpassing 88.50% for each performance metric. These results indicate that the proposed model achieved outstanding results over existing deep learning models.

Figures 8, 9, 10 present a visualization of simulation results for the proposed model, Figs. 11, 12, 13 present a visualization of simulation results for the MobileNet Model, Figs. 14, 15, 16 present a visualization of simulation results for the MobileNet-V2 Model, Figs. 17, 18, 19 shows a visualization of simulation results for the VGG-16 Model, Figs. 20, 21, 22 present a visualization of simulation results for the VGG-19 Model on the “HAM-10000” skin cancer dataset. Similar Figs. 23, 24, 25 present a visualization of simulation results for the Resnet-152v2Model on the “HAM-10000” skin cancer dataset. Based on the analysis of the results, it’s clearly proven that the proposed model has better simulation outcomes than existing models. Existing models such as MobileNet, VGG-16, MobileNet-V2, ResNet-152v2, and VGG-19 did not perform as well as the Hybrid U-Net, and Improved MobileNet-V3 model with hyperparameter optimization did on the "HAM-10000 Melanoma Skin Cancer dataset." There are several reasons for this.

- *Architecture of U-Net* The U-Net architecture has gained recognition for its efficacy in the field of image segmentation, particularly in tasks related to skin cancer detection, where the objective is to delineate skin tumours from the tissue that surrounds them accurately. The U-Net's capacity to effectively capture intricate details and delineations contributes to enhanced feature extraction and superior classification outcomes.
- *Improved MobileNet-V3* MobileNet-V3 is a CNN architecture that has been specifically developed to prioritize both efficiency and accuracy. The enhanced iteration of the dataset takes into account alterations and refinements that render it better suited for the unique attributes of the skin malignancies dataset.
- *Feature extraction by Hybrid Model* The hybrid models were created by integrating the advantageous features of both U-Net and MobileNet-V3. The provided system offers precise segmentation masks, whereas MobileNet-V3 efficiently extracts features from the segmented regions. The integration of these characteristics enhances the model's comprehension of images related to skin cancer.
- *Optimization of Hyperparameter* The utilization of hyperparameter optimization techniques enables the refinement of the model's parameters and structural decisions, specifically tailored to the skin cancer dataset. In comparison with models with standard hyperparameters, this led to better performance and broad applicability.

#### Ablation analysis

We performed ablation research to determine the effect of critical parameters in the proposed skin cancer analysis model. To check the proposed model performance, we have conducted an ablation analysis with various conditions, which include (a) with and without Hybrid U-Net, (b) with and without MobileNet-V3, (c) with and without Augmentation, (d) with and without Batch Normalization, (e) with and without Hyperparameters. After methodically removing and testing with every single subsequent component eliminated, the following observations were found.

**Figure 26:**
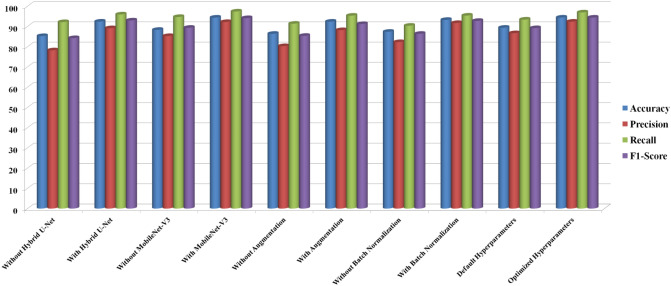
Comparative analysis of ablation analysis for different parameters on skin dataset.

Various factors on the skin dataset were compared in the ablation analysis, as shown in Table 8 and Fig. 26. For example, when it came to identifying complicated characteristics inside skin lesions, performance metrics dropped when the Hybrid U-Net was not used. There has been a significant gain in accuracy, suggesting that the Hybrid U-Net is vital in lowering the model's false positive rate and improving its capacity to detect cancerous tumours accurately. Improving MobileNet-V3 is crucial for efficient feature extraction since its absence significantly reduced total accuracy. There is strong evidence from the improved accuracy that Improved MobileNet-V3 helps cut down on false positives.

**Table 8:** Comparative analysis of ablation analysis for different parameters on skin dataset.

Metric	Hybrid U-Net		MobileNet-V3		Augmentation		Batch Normalization		Hyperparameters	
	Without Hybrid U-Net	With Hybrid U-Net	Without MobileNet-V3	With MobileNet-V3	Without Augmentation	With Augmentation	Without Batch Normalization	With Batch Normalization	Default Hyperparameters	Optimized Hyperparameters
Accuracy	85.25	92.4	88.32	94.38	86.38	92.38	87.37	93.25	89.37	94.39
Precision	78.2	89.14	85.27	92.2	80.28	88.18	82.27	91.71	86.67	92.37
Recall	92.15	95.93	94.69	97.38	91.37	95.33	90.37	95.37	93.4	96.92
F1-Score	84.24	92.96	89.35	94.13	85.37	91.2	86.38	92.75	89.17	94.37

Results showed that model resilience was reduced in the absence of data augmentation, highlighting the need for dataset augmentation for improved generalizability. Increases in accuracy and F1-score demonstrate how data augmentation enhances the model's capacity to categorize cancerous tumours accurately. A decrease in overall model performance and a slowdown in training convergence were the outcomes of removing batch normalization layers. Improving the model's capacity to distinguish between benign and malignant lesions and stabilizing the training process are both achieved using batch normalization, as seen by the significant gains in accuracy and F1-score. We found that the model's sensitivity to distinguishing between benign and malignant lesions is greatly affected by the ideal values of the hyperparameters. These enhanced precision and accuracy show how critical hyperparameter adjustment is for getting the most out of a model. All of the steps and parts of our skin cancer diagnostic methodology are vital, as these results show. In addition to shedding light on the model, the ablation research provides valuable information for making future upgrades and enhancements. Here are some practical ways to improve the model's diagnostic accuracy and reliability: observe the changes in performance measures.

## Conclusion and future works

The proposed research integrates Hybrid U-Net and Improved MobileNet-V3 features to formulate a new hybrid model for skin cancer detection. Our model, showcasing superior diagnostic accuracy, emphasizes the pivotal roles of Hybrid U-Net and Improved MobileNet-V3 through a comprehensive assessment involving an ablation study and a comparison with state-of-the-art models. Beyond delivering a high-performing model, the study offers insightful directions for future research, contributing to the advancements in automated skin cancer detection. The observed enhancements, coupled with our competitive standing against current models, suggest a potentially significant therapeutic impact. According to the authors, this study introduces new possibilities for research and development in medical image analysis, promising faster and more accurate skin cancer diagnostics.

This research introduces an innovative skin cancer detection method incorporating U-Net and MobileNetV3 architecture, employing hyperparameter optimization strategies. By combining advanced deep learning techniques and meticulous parameter optimization, the result is a highly accurate and efficient system for diagnosing skin cancer. This work significantly improves skincare and medical services by providing a valuable tool for skin specialists and doctors to enhance the timely detection of skin malignancies and improve patient outcomes. The proposed model is compared with existing models, namely MobileNet, VGG-16, MobileNet-V2, Resnet-152v2, and VGG-19 on the "HAM-10000 Melanoma Skin Cancer dataset".

To fine-tune the model's hyperparameter, we employ sophisticated optimization methods such as the Bayesian optimization method using the pre-trained CNN architecture MobileNet-V3. Empirical findings demonstrate that the proposed optimized hybrid MobileNet-V3 model outperforms existing skin cancer detection and segmentation techniques, achieving high precision (97.84%), sensitivity (96.35%), accuracy (98.86%), and specificity (97.32%). The enhanced performance of this research can lead to timelier and more precise diagnoses, potentially contributing to life-saving outcomes and mitigating healthcare expenditures. The developed model holds significant potential in terms of clinical relevance, as suggested by its precision and recall scores, making it a valuable tool for dermatologists in the early detection of skin cancer. The elevated accuracy level presents a potential avenue for mitigating misdiagnoses and improving patient outcomes.

In the proposed model, computational demands during training and inference may arise as a consequence of integrating two complicated designs. This limitation should be taken into account, particularly in situations when resources are limited. Also, interpretability is a common issue for deep learning models like the proposed model. In clinical situations, the model's interpretability is limited because, despite its good performance, comprehending the precise elements underlying the classification judgments might be difficult.

In order to reduce computing complexity, future studies should concentrate on improving the model. To improve the practicality of deployment, it may be worthwhile to investigate model compression approaches or lightweight designs. Solving problems with interpretability might be crucial. Healthcare providers may gain confidence and knowledge of the model's decision-making process with the use of explainable AI approaches. Future research in the area of automated skin cancer detection will be guided by this balanced approach, which adds to the current conversation in the field. Future research should explore incorporating supplementary clinical data, developing interpretability methods tailored for medical professionals, and addressing the ethical implications associated with implementing artificial intelligence models for skin cancer diagnosis. Our commitment to innovation remains central as we aim to make a significant impact within dermatology. While the proposed model exhibits exemplary performance on the HAM-10000 dataset, it is crucial to recognize that real-world medical records may show unforeseen variations. Additional validation is necessary to extend the model's capabilities to datasets and situations in healthcare that have not been previously encountered.

## Data Availability

The data supporting the research results of the present research can be obtained by contacting the corresponding author.

## Abbreviations

DL: Deep learning
ISIC: International skin imaging collaboration
ADAM: Adaptive moment optimization algorithm
FC: Fully connected
DL: Deep learning
CNN: Convolution neural network
BN: Batch normalization
SVM: Support vector machine
ReLu: Rectified linear unit
TL: Transfer learning
